# Personalizing Human-Agent Interaction Through Cognitive Models

**DOI:** 10.3389/fpsyg.2020.561510

**Published:** 2020-09-24

**Authors:** Tim Schürmann, Philipp Beckerle

**Affiliations:** ^1^Work and Engineering Psychology Research Group, Department of Human Sciences, Technical University of Darmstadt, Darmstadt, Germany; ^2^Elastic Lightweight Robotics, Department of Electrical Engineering and Information Technology, Robotics Research Institute, Technische Universität Dortmund, Dortmund, Germany; ^3^Institute for Mechatronic Systems, Mechanical Engineering, Technical University of Darmstadt, Darmstadt, Germany

**Keywords:** personalization, cognitive modeling, human-agent interaction, behavior prediction/generation, interaction adaption

## Abstract

Cognitive modeling of human behavior has advanced the understanding of underlying processes in several domains of psychology and cognitive science. In this article, we outline how we expect cognitive modeling to improve comprehension of individual cognitive processes in human-agent interaction and, particularly, human-robot interaction (HRI). We argue that cognitive models offer advantages compared to data-analytical models, specifically for research questions with expressed interest in theories of cognitive functions. However, the implementation of cognitive models is arguably more complex than common statistical procedures. Additionally, cognitive modeling paradigms typically have an explicit commitment to an underlying computational theory. We propose a conceptual framework for designing cognitive models that aims to identify whether the use of cognitive modeling is applicable to a given research question. The framework consists of five external and internal aspects related to the modeling process: research question, level of analysis, modeling paradigms, computational properties, and iterative model development. In addition to deriving our framework from a concise literature analysis, we discuss challenges and potentials of cognitive modeling. We expect cognitive models to leverage personalized human behavior prediction, agent behavior generation, and interaction pretraining as well as adaptation, which we outline with application examples from personalized HRI.

## Introduction

Contemporary approaches highlight the relevance of personalization in human-agent interaction (HAI). For example, e-commerce applications that use web personalization to create product deals and recommendations for users traditionally enjoy persistent research interest (Salonen and Karjaluoto, 2016). However, personalization has also long since branched out from e-commerce to further areas of human-computer interaction (HCI), such as activity recognition (Sztyler and Stuckenschmidt, 2017; Zunino et al., 2017; Siirtola et al., 2019), body part tracking (Tkach et al., 2017), assisted driving (Hasenjäger and Wersing, 2017), and human-robot interaction (HRI; Clabaugh and Matarić, 2018; Collins, 2019; Irfan et al., 2019). Although user experience of personalized services is positively influenced by overtness and transparency (Chen and Sundar, 2018; Dolin et al., 2018), personalization is not universally appreciated due to concerns over users’ loss of information privacy (Alatalo and Siponen, 2001; Chellappa and Sin, 2005; Awad and Krishnan, 2006; Schneider et al., 2017; Ku et al., 2018).

As Graus and Ferwerda (2019) argue, personalization is typically achieved by a system adapting to data-driven inference about users based on their previous behaviors. Their study posits that a theoretically motivated approach may lead to two benefits over a purely data-driven model: reducing the need for extensive data analysis and potentially generating new insight regarding the appropriateness of a given theory. The sentiment for more theory-driven approaches in data analysis is also shared by Plonsky et al. (2019) and Bourgin et al. (2019). Both articles highlight the improved prediction of human decisions by machine learning models after implementing variants of behaviorally relevant psychological theories. Bourgin et al. (2019) specifically make the case for pretraining machine learning models with data simulated by cognitive models. Cognitive models refer to the instantiation of a theory that relates to one or more cognitive functions and tries computationally to replicate them. Due to this, cognitive modeling is routinely used synonymously with computational modeling (Sun, 2008a). In previous research, the application of cognitive models has helped to explain or recontextualize several empirically established psychological phenomena (Adams, 2007; Körding et al., 2007; Vul et al., 2014; Srivastava and Vul, 2017). It is routinely argued that the advantage of cognitive models over, for example, verbal-conceptual or data-driven statistical models lie in the need to translate a theoretical framework into a computational system, leaving less freedom for interpretation (Sun, 2008a; Stafford, 2009; Murphy, 2011; Farkaš, 2012). In contrast to cognitive models, verbal-conceptual models define no formal relationship between concepts in a mathematical sense, and statistical models use mathematical equations to describe the relationship between concepts but do not require the translation into a computational system. Sun (2008a) notes that statistical models “may be viewed as a subset of computational models, as normally they can readily lead to computational implementations […].”

**Figure 1:**
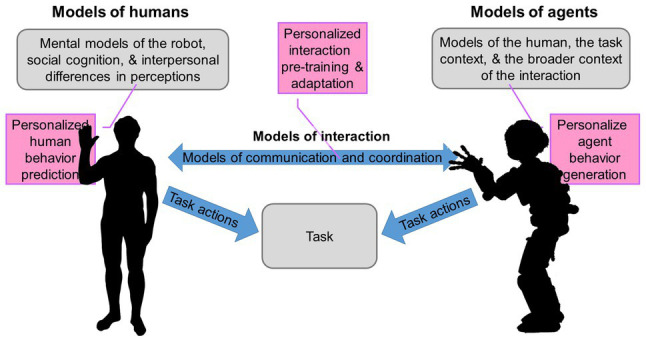
Cognitive human-robot interaction (HRI) as presented and discussed by Mutlu et al. (2016) as an example of human-agent interaction (HAI) (blue and gray). Various interaction challenges might be tackled applying cognitive models and exhibit strong potential for personalization (magenta). For instance, human behavior prediction (models of humans), interaction pretraining and adaptation (models of coordination), and generating agent behavior from human models (models of agents).

As Plonsky et al. (2019) and Bourgin et al. (2019) show, involving cognitive models in human behavior prediction as outlined in Figure 1 increases predictive performance. It is reasonable to assume that a similar performance increase can be expected by incorporating cognitive models into the data-analytic inference required for personalization (Graus and Ferwerda, 2019) and in (personalized) HRI (Collins, 2019; Cross et al., 2019; Fischer and Demiris, 2019; Prescott et al., 2019). Following from this, this article discusses challenges and potentials of cognitive models focusing on user-specific effects and proposes a conceptual framework for (personalized) model development in Section “A Conceptual Framework for Designing Cognitive Models.” Subsequently, we discuss the HRI application examples from Figure 1 in detail and analyze common pitfalls in Section “Application Examples and Pitfalls.” Section “Conclusion” concludes by discussing connections of personalization and cognitive modeling and outlining directions for future research.

## A Conceptual Framework for Designing Cognitive Models

**Figure 2:**
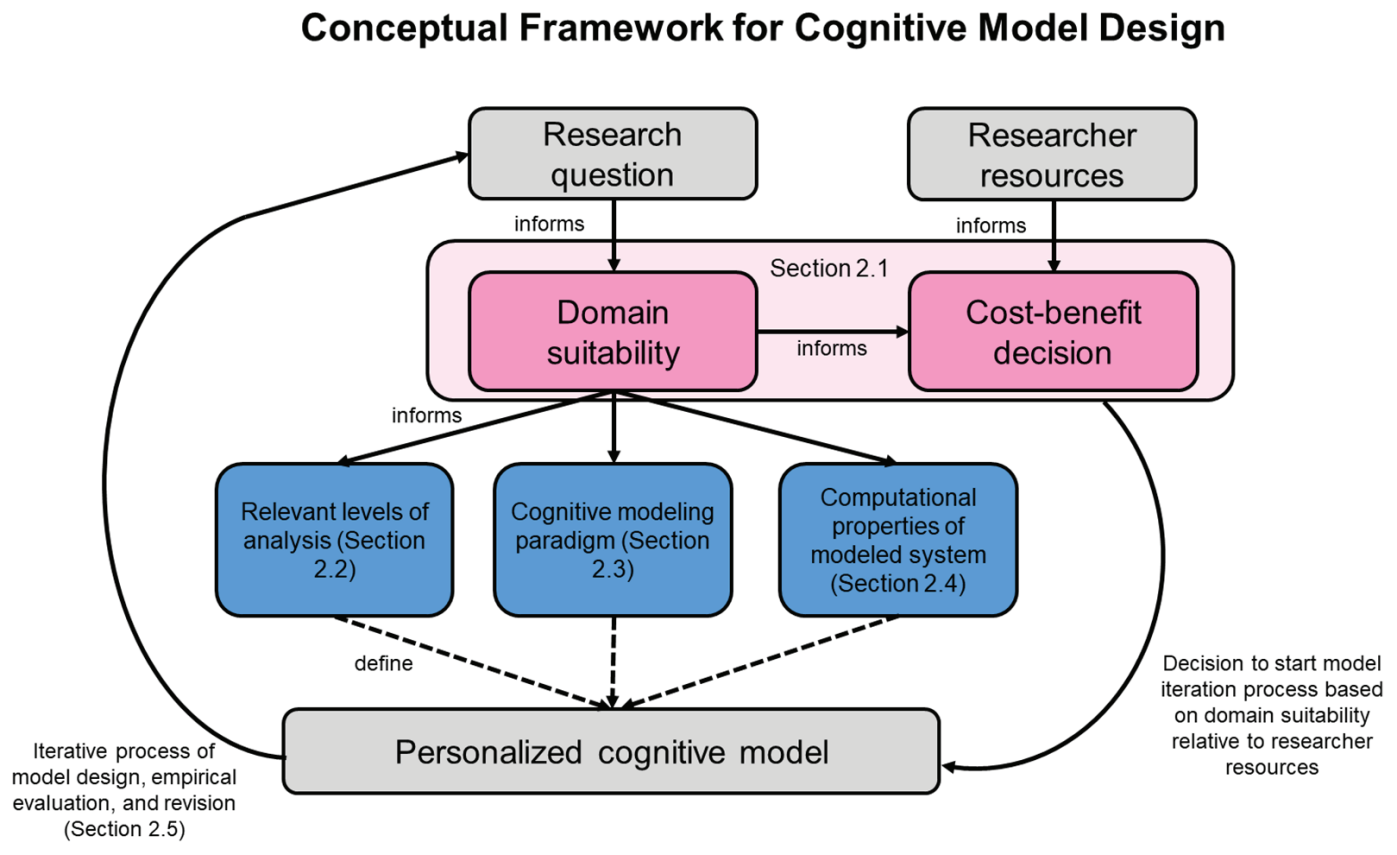
Conceptual framework for cognitive model design. External aspects are displayed in gray, and pink indicates initial considerations that inform the specific design decisions, which are themselves colored blue. The framework is used by evaluating domain suitability and available resources to reach a decision on whether to initiate the modeling process. Then, model-related aspects are defined and the resulting model is empirically evaluated. If necessary, an iterative process of model improvement is started.

We present a conceptual framework to consider model-related and external aspects when designing cognitive models, following the definition of conceptual frameworks given by Imenda (2014). As an inductive synthesis of existing theoretical and empirical insights, the proposed framework highlights important considerations with regard to cognitive modeling, specifically for researchers new to the method. Figure 2 provides a schematic representation of the framework components and their interactions, which are presented and discussed in the remainder of the paper. Researchers applying the framework start by evaluating the domain suitability of the research question and make a cost-benefit decision based on the suitability and available resources. Given a positive evaluation, they define the model-related aspects that constrain the actual cognitive model. Based on the model’s performance in predicting empirical data, there may be a need to revise the model design and evaluate again or use the available model to investigate the research question.

### Research Questions and Resources

An initial threshold regarding the application of cognitive models is the considered research question, i.e., questions related to cognitive functions. Sun (2008a) identifies several cognitive functions that can be approached by cognitive modeling: motivation, emotion, perception, categorization, memory, decision making, reasoning, planning, problem-solving, motor control, learning, metacognition, language, and communication. If included in a theory, these cognitive functions may suggest a computational view toward human behavior and may, therefore, benefit from cognitive modeling. Because many other types of models lack the precision derived from formalized model definition (Murphy, 2011), cognitive models can help to understand the functions suggested by Sun (2008a). The aforementioned cognitive functions develop highly interindividually, and cognitive states in a given situation are difficult to generalize. Therefore, Lee (2011) highlights the importance of accounting for individual differences in the execution of cognitive functions and proposes hierarchical cognitive modeling as a way to do so. Using cognitive models to estimate and then maintain a representation of the motivational, emotional, or other cognitive states of individuals allows an interactive system to adjust its behavior and, accordingly, may help to personalize user experience with HAI systems (Schürmann et al., 2019). It is still debated whether statistical or verbal-conceptual models (Sun, 2008a; Çelikok et al., 2019; Guest and Martin, 2020) provide the required conceptual precision to shed light on the underlying theory. In our opinion, the application of cognitive modeling is less beneficial for research questions that do not directly deal with the cognitive functions mentioned above or research questions that lack established assumptions about how these cognitive functions work. As depicted in Figure 2, the suitability of cognitive modeling is determined based on the related cognitive functions.

A second important requirement of applying cognitive modeling relates to resources available to the researcher, e.g., programming capabilities. To our knowledge, there is no software solution available that allows for cognitive modeling design without programming expertise. As Addyman and French (2012) point out, even simulation environments such as ACT-R (Ritter et al., 2019) are often of little use to researchers without programming experience. Although most programming languages should be capable of the required mathematical operations, high-level languages focusing on statistics and providing function libraries, e.g., R, Python or Matlab, can strongly simplify cognitive modeling. In our framework, programming resources and the suitability of the research question inform a cost-benefit decision that indicates whether the development of a cognitive model should be started (see Figure 2).

### Relevant Levels of Analysis

Marr (1982) defines three levels of analysis on which the study of cognitive systems is most commonly based. These levels do not fall into a strict hierarchy but can be understood as complementary descriptions of a cognitive system from equally important perspectives. The first step when applying our framework is to clarify to which levels of analysis the cognitive model in question may be connected (left path in Figure 2). Answering this question provides the researcher with constraints for further modeling steps. The computational level includes the content of computations that a cognitive system, irrespective of being human or artificial, executes. This includes the logic and structure of the problem or task that a cognitive system attempts to solve. The algorithmic level contains information about the processes and representations that describe the computation. Last, the implementational level deals with the biological or artificial realization in physical hardware. Zednik and Jakel (2014) paraphrase this categorization of levels of analysis; the computational level specifies what a system is doing and why it is doing it; the algorithmic level specifies the how; and the implementational level specifies the where. Over time, researchers have suggested adding layers to the levels of analysis (Griffiths et al., 2015) or adjusting models so that they are defined on more than one level of analysis (Griffiths et al., 2012; Vul et al., 2014).

Applying cognitive modeling to a given research question includes identifying the levels of analysis that are most relevant or applicable, i.e., which level of analysis is required to describe the given problem. For example, Griffiths et al. (2008) argue that Bayesian cognitive modeling is more suitable for problems of inductive inference than for predicting human behavior due to the mathematical structure of Bayes’ rule. Outlining the scope of the problem that the cognitive system is expected to solve leads, in the authors’ experience, to an intuitive restriction of applicable levels of analysis. If one can assume that all individuals solve the same cognitive problem, the level of analysis chosen is not something to be personalized but rather a modeling choice that determines the possible dimensions of personalization in subsequent steps.

### Selection of Cognitive Modeling Paradigms

Considering the identified cognitive problem, several modeling paradigms may present themselves, each with their own potential for personalization. These candidate paradigms are routinely, but not necessarily, defined on the same level of analysis (Marr, 1982) as the cognitive problem they approach. One could argue that the more levels covered by a model’s predictions, the more complete the understanding of a phenomenon is. For example, instead of providing a predicted response to a choice problem, a model can also provide an estimate of predicted reaction time required to respond to the choice problem. Although covering multiple levels has the potential to provide new insights, a research question may not yet include any reasonable assumptions about reaction times so that the required additional specifications of a prediction time model could be theoretically under-constrained. Additionally, covering Marr’s levels completely may not be necessary for all research problems; e.g., cognitive algorithms might be powerful extensions to existing robotic platforms.

Depending on the relation to cognitive functions and levels of analysis (Marr, 1982), an appropriate cognitive modeling paradigm should be selected (middle path in Figure 2). Sun (2008b) identifies the following paradigms: connectionism, Bayesianism, dynamical systems approaches, declarative or logic-based models, and cognitive architectures. All these paradigms allow for free parameters that govern individual model behavior and, hence, allow for personalization by parameter fitting. Moreover, the paradigms have soft boundaries, and mathematical representations of specific cognitive processes overlap (Roe et al., 2001; Fard et al., 2017). The number of free parameters in cognitive models can, however, cause overfitting as discussed in Section “Application Examples and Pitfalls.” Therefore, we advise readers to approach the selection of cognitive modeling paradigms driven by their research question’s underlying theory: Assuming interest in whether human choices satisfy criteria of rationality, juxtaposing a Bayesian model as a proxy for computational rationality against a heuristic model of violations against computational rationality is a suitable approach. As another example, a research question could concern specific neurological processes and, therefore, be compatible with modeling paradigms with an extension to implementational level of Marr (1982), i.e., the neural hardware.

### Computational Properties of the Modeled System

As previously outlined, there are no general indications to select modeling paradigms or covering levels of analysis (Marr, 1982). Therefore, it appears suitable to consider the required computational properties to adequately account for the modeled behavior. This consideration is captured in the third path of our framework (right path in Figure 2). Calder et al. (2018) outline some computational properties: deterministic and nondeterministic (representing behavior by probabilities) models, static and dynamic (representing temporal effects) models, discrete or continuous models, and models based on individuals or populations. If a model is deterministic, it always produces the same behavior given the same input, and a nondeterministic model produces the behavior based on an internal probability. A static model has no inherent concept of time, and a dynamic one does. Discrete models represent their components in steps or levels, and continuous models use representations that are smooth. We posit that, as different models can be used to describe the same human behavior, they likely share similar properties. For HAI research, we assume that individual-focused models that are nondeterministic in nature to represent the probabilistic aspects of human choice and perception (Körding et al., 2007; Rieskamp, 2008) appear beneficial to provide accurate predictions of the target behavior. If the behavior of interest is human choice and perception, we consider the focus on individuals and non-determinism as necessary properties of a model. Whether a model operates discretely or continuously and whether it is static or dynamic may depend on the research question or cognitive function.

A principled way of drawing inference about a cognitive model’s parameters on intra‐ and inter-individual levels comes in the form of hierarchical cognitive modeling (Lee, 2011). Once required computational properties have been defined, this hierarchical approach considers an individual’s model parameters to be sampled from a population-wide distribution of parameters. In this way, both inter‐ and intra-individual variations in the behavior of human users can be respected by HAI systems with hierarchical modeling levels, thus allowing for personalization of the cognitive model.

### Iterative Model Development, Evaluation, and Revision

Our proposed framework considers the external aspects, and settling on specific decisions regarding model development should result in a functioning and testable cognitive model. Evaluating the resulting model against empirical evidence or competing models, however, may show a gap between model predictions and observed behavior, depending on the specific nature of the research question. This suggests an iterative process of model development, evaluation, and revision, which provides the opportunity to reassess whether a specific combination of levels of analysis, modeling paradigm, and computational properties suits the research question. Murphy (2011) highlights that certain aspects of human behavior might not be understood well enough to justify using a formalized theory and a cognitive model building on said theory. However, an indication of whether we know enough or not is the repeated reference to formalized theories of cognitive functions in the literature. An applied example of this can be found in research about human user behavior in online services. Schürmann et al. (2020) conduct a secondary literature review in which they reanalyze existing review data concerning the frequency of references to computational-level theories, the frequency of interpretations of statistical model results as computational, and the frequency of actual computational implementations. References to formalized theories are found in 44.2% of the investigated literature, and results of statistical models are interpreted in a computational manner in 33.3% of cases. However, the prevalence of cognitive modeling implementations is low at 5% (Schürmann et al., 2020). Accordingly, it seems that information is sufficient to warrant statements about the cognitive functions of online users. An iterative model development process can then close in on suitable specifications such as the level(s) of analysis, modeling paradigm, and computational properties required to adequately describe a target behavior. To implement personalization, the formalization of inter-individual differences appears necessary. Although these could be represented as parameter differences in statistical models (Sun, 2008b) as well, cognitive models are potentially leading to improved understanding and theories of cognitive functions.

## Application Examples and Pitfalls

Before highlighting application examples and pitfalls of cognitive modeling in HAI with regard to personalization, it is necessary to define applications of cognitive models. We differentiate between three applications of cognitive models as outlined in Figure 1: (1) using models of human agents to understand decisional or perceptual processes to improve predictions of the agent’s behavior, (2) modeling human behavior to pretrain and adapt interaction, e.g., to monitor users’ preferences, and (3) generating behavior of an artificial agent based on a cognitive model of human behavior.

The agent of interest may be a humanoid robot, a chat bot, or any type of system that might benefit from generating its own behavior in a human-like manner. In the remainder of this section, we focus on interaction between humans and humanoid robots as shown in Figure 1 because we deem it a striking and very graspable exemplary case. Here, robotic agents may use cognitive models to predict human interactions, but they may also control their own sensorimotor behavior by use of such a cognitive model. The benefit of applying cognitive approaches lies in the potentially realistic imitation of human behavior and can foster both psychological research and the development of humanoid robots (Asada et al., 2009; Hoffmann et al., 2010; Schillaci et al., 2016; Prescott et al., 2019; Schürmann et al., 2019). Through fitting free parameters to interindividual differences, behavior prediction and generation can be personalized rather straightforwardly. Combining the idea of human and robot models with the approach of Bourgin et al. (2019) to pretrain contemporary machine learning models with cognitive models, we argue that humanoid robots could produce more human-like sensorimotor behavior that fosters interaction and adapts to the human partner. Considering the example of a human-robot handshake, cognitive models could be used to predict a user’s movement selection (behavior prediction) and control the humanoid’s motion execution (behavior generation) and also to align the robot’s actions to the human partner, spatially and temporally (interaction adaptation; Wang et al., 2013; Vogt et al., 2017).

Pitfalls of applying cognitive models to HAI are generally similar to other domains. The advantage of higher formalization and predictive precision comes at the price of having to communicate programming-related and mathematical concepts to audiences that may be used to verbal-conceptual theories. Additionally, development, maintenance, and publication of model code represent considerable challenges compared to less computationally sophisticated methods. When programming a model, researchers need to be aware of the relation between the number of free parameters in a model and the danger of overfitting (Farrell and Lewandowsky, 2018). Specifically, within the context of personalization, the danger of overfitting individual differences lies in the loss of generalization so that the prediction of new, previously unseen users would be initially poor. Recently, researchers have noted that cognitive models run the risk of having fundamental aspects adjusted after empirical data have been observed for the purpose of increasing the fit to the data (Lee et al., 2019). This pitfall should be given special consideration when applying cognitive models to scenarios of personalization.

## Conclusion

Cognitive modeling has strong potential in general and personalized HAI. We recommend considering the given conditions, especially whether the interactive task deals with the inter-individual aspects of cognitive functions. The conceptual framework proposed in this article helps to determine which cognitive function is of relevance and which cognitive modeling paradigm satisfies the required computational properties and serves for personalization as well as whether formal theories of cognition exist. Moreover, using the framework in HAI systems may help to discern whether a cognitive model could be used to predict human behavior, to pretrain and adapt interaction, and/or to generate the behavior of an artificial agent in a personalized fashion (see Figure 1).

Although not too commonly used, personalized HAI can be realized with many contemporary modeling paradigms through fitting free parameters or even online adaptation of model structures. We outline conditions that, when met, put cognitive modeling in a strong position to provide insights that cannot be provided by otherwise prominent statistical models. As Graus and Ferwerda (2019) suggest, theory-driven models benefit from a reduced need for extensive data analysis. Whether this holds true for cognitive models, which are notorious for their quickly rising number of free parameters, remains to be seen. As a second benefit, the generation of new insights seems particularly important with respect to cognitive models. Aside from theory-driven personalization, an added value of cognitive modeling in HAI stems from its practical application, e.g., in humanoid robot development. Here, improvement of the interaction with particular human users would not only result directly from the representation of inter-individual differences, but also from a general approximation of human behavior. First, human-like, e.g., less precise but more versatile, robot movements have been shown to improve the perceived interaction quality (Pan et al., 2019). Second, cognitive modeling has been successfully used for pretraining machine learning models (Bourgin et al., 2019), which increases learning efficiency and has strong potential to foster distinct progress in personalizing interaction.

Applying the proposed framework can clarify the relation between external and internal aspects of cognitive modeling and, especially, support first-time users. Future research should elaborate the conceptual framework in empirical HAI studies; focusing the purposes outlined in Figure 1 will help to improve personalized interaction.

## Author Contributions

All authors listed have made a substantial, direct and intellectual contribution to the work, and approved it for publication.

### Conflict of Interest

The authors declare that the research was conducted in the absence of any commercial or financial relationships that could be construed as a potential conflict of interest.
